# The Transaldolase, a Novel Allergen of *Fusarium proliferatum*, Demonstrates IgE Cross-Reactivity with Its Human Analogue

**DOI:** 10.1371/journal.pone.0103488

**Published:** 2014-07-30

**Authors:** Hong Chou, Keh-Gong Wu, Chang-Ching Yeh, Hsiao-Yun Tai, Ming F. Tam, Yu-Sen Chen, Horng-Der Shen

**Affiliations:** 1 Department of Medical Research, Taipei Veterans General Hospital, Taipei, Taiwan, R.O.C.; 2 Department of Pediatrics, Taipei Veterans General Hospital and National Yang-Ming University, Taipei, Taiwan, R.O.C.; 3 Department of Obstetrics and Gynecology, Taipei Veterans General Hospital, Taipei, Taiwan, R.O.C.; 4 Department of Biological Sciences, Carnegie Mellon University, Pittsburgh, Pennsylvania, United States of America; University of Rochester Medical Center, United States of America

## Abstract

*Fusarium* species are among airborne fungi and recognized as causative agents of human atopic disorders. However, *Fusarium* allergens have not been well characterized and the lack of information limits clinical diagnosis and treatment of fungal allergy. The purpose of this study is to identify and characterize important allergens of *F. proliferatum*. IgE-reacting *F. proliferatum* components were identified by immunoblot using serum samples from patients of respiratory atopic diseases. Characterization of allergens and determination of IgE cross-reactivity were performed by cDNA cloning, then homologous expression and immunoblot inhibition studies. We identified nine different *F. proliferatum* components that can be recognized by IgE antibodies in 17 (28%) of the 60 atopic sera tested. Components with molecular masses of about 43, 37.5 and 36.5 kDa with IgE-binding frequencies of about 88, 47 and 53%, respectively, were considered as important allergens of *F. proliferatum*. The 37.5 kDa IgE-binding component was putatively considered as a transaldolase protein of *F. proliferatum*. The full-length cDNA of *F. proliferatum* transaldolase was subsequently cloned. It encodes an open reading frame of 312 amino acids and has sequence identifies of 73 and 61%, respectively, with *Cladosporium* and human transaldolases. The purified recombinant *F. proliferatum* transaldolase can inhibit the IgE-binding against the 37.5 kDa component of *F. proliferatum* and the transaldolase allergen from *Cladosporium cladosporioides*. More importantly, the recombinant *F. proliferatum* transaldolase can inhibit IgE-binding against human transaldolase in a concentration-dependent manner. Thus, a novel and important *F. proliferatum* transaldolase allergen was identified. In addition to IgE cross-reactivity between the *Fusarium* and the *Cladosporium* transaldolase allergens, IgE cross-reactivity between the *Fusarium* and the human transaldolases also exists and might contribute to atopic manifestations in the absence of exogenous allergen exposure.

## Introduction

The prevalence of human atopic diseases including allergic rhinitis and asthma is increasing during recent decades [1]. Sensitization to molds which are ubiquitous in our environment has been reported to be close to 80% of asthmatic patients [2]. Hence, it is important to characterize fungal allergens and subsequently provide a basis for better diagnosis and treatment of fungal allergy [2]–[4]. However, fungal allergens are difficult to be defined since multitudinous factors contribute to the results obtained [3], [5]. Due to these inherent difficulties, the manufacturing of standardized and high quality fungal allergen extracts is not available in the United States [4]. It highlights the necessity of characterizing important fungal allergens [2]–[4].

*Cladosporium* species are the dominant airborne spores worldwide [3]. In addition, *Alternaria*, *Aspergillus*, *Penicillium* and *Fusarium* species are also airborne fungi in many areas including Taipei [6]–[8]. *Cladosporium* and *Alternaria* are clinically important causative allergenic agents for patients sensitive to fungi [2]. *Fusarium* fungus can emit large amount of spores in rainy or humid enrivonment [9]. Thus, it is of interest to study the relevance of *Fusarium* fungus to allergic sensitization. Chang et al. [10] tested a list of 54 air-borne allergens in 66 bronchial asthma patients in the Taipei area, and 20 (30%) of the patients showed positive skin reaction to *Fusarium* extracts. O’Neil et al. [11] found that among 69 atopic individuals tested in United States, 17 (24%) of the patients had positive skin reactions to an extract of *F. solani*. Stroud et al. [12] reported that reactivity to fungi was found in 65% of chronic rhinitis patients and reactions to *Fusarium* (58%), *Alternaria* (39%) and *Pullularia* (38%) were particularly common. Using in-house extracts for EAST and immunoblot experiments, Hoff et al. [13] detected *F. culmorum* specific IgE antibodies in 23 (44%) of 52 subjects with suspected mould allergy in Europe. In India, skin prick tests with 60 allergens were performed on 48 patients with naso-bronchial allergy and results indicated that *Aspergillus fumigatus*, *A. flavus*, *Alternaria teneis* and *F. solani* were common fungal allergens [14]. In Greece, Gonianakis et al. [15] found that among 571 patients, 42% showed dermal positivity to allergens derived from *Alternaria, Cladosporium, Fusarium, Aspergillus*, and *Mucor*. Thus, there is a worldwide indication that *Fusarium* fungus may play a role in clinical allergy. However, our knowledge about allergens of this airborne *Fusarium* fungus is still quite limited [13], [16] and standardized *Fusarium* extracts for clinical diagnostics are lacking.

IgE cross-reactivity is an important component of fungal sensitization and could contribute significantly to allergy manifestation [17]. Thus, in addition to the identification and characterization of fungal allergens, it is important to delineate IgE cross-reactivity between allergens from different fungal species and even more importantly, between fungal allergens and their human analogues. Previously, we have identified important IgE cross-reactive pan-serine protease fungal allergens from prevalent *Penicillium* and *Aspergillus* species [18]. Recently, in addition to serine proteases, the transaldolase has also been identified as a significant and IgE cross-reactive allergen family of *Cladosporium* and *Penicillium* species [19]. The purpose of this study is to identify and characterize allergens of *Fusarium* species. Our results show that the 37.5 kDa transaldolase is a novel and important allergen of *F. proliferatum* (Fus p 4.0101). In addition, Fus p 4.0101 demonstrated IgE cross-reactivity with the transaldolase allergen from *C. cladosporioides* (Cla c 14.0101) and, interestingly, with the human transaldolase.

## Materials and Methods

### Serum samples

The sixty serum samples used in this study were obtained from the Biobank at the Taipei Veterans General Hospital. All these serum samples were obtained from respiratory atopic patients (allergic rhinitis and/or atopic asthma) who attended the allergy clinics of the Taipei Veterans General Hospital and were stored in aliquots at −80°C. This study has been approved by the Institutional Review Board of the Taipei Veterans General Hospital.

### Crude extracts of *F. proliferatum*

*F. proliferatum* strain BCRC 30972 was used in this study. It was isolated from the air of Taiwan and provided by the Food Industry Research and Development Institute, Hsinchu, Taiwan. It was cultured in a CYB medium without agitation at 26°C for five days. The CYB medium contains yeast carbon base (Difco Laboratories, Detroit MI, USA; 11.7 g/L), glucose (Mallinckrodt Baker, Inc., Phillipsburg, NJ, USA; 10 g/L) and casein enzymatic hydrolysate (Sigma Chemical Co., St. Louis, MO, USA; 10 g/L). Crude extracts of *F. proliferatum* were prepared essentially as described [19], [20]. The protein content of crude fungal extracts was determined with a dye-binding assay according to the manufacturer’s instructions (Bio-Rad, Richmond, CA, USA).

### Sodium dodecyl sulfate-polyacrylamide gel electrophoresis (SDS-PAGE) and immunoblotting

Proteins in the crude fungal extractions or purified recombinant proteins were separated by SDS-PAGE [19], [20] then transferred electrophoretically onto polyvinylidene difluoride (PVDF) membranes (0.45 µm, Millipore, Bedford, MS, USA). Protein components reacting against human IgE antibodies were determined as described [19], [20]. The membranes were blocked with 1% skimmed milk and incubated with serum samples for 16 h at 4°C. The membranes were washed, incubated with alkaline phosphatase-conjugated monoclonal anti-human IgE antibodies (Pharmingen, San Diego, CA, USA) then developed with enzyme substrates essentially as described [19], [20]. Serum samples from a non-atopic healthy individual and two house dust mite (*Dermatophagoides pteronyssinus*)-sensitized atopic individuals were used as controls.

### cDNA Cloning

The cDNA encoding the *F. proliferatum* transaldolase was isolated with polymerase chain reactions (PCR) using an AffinityScript Multiple Temperature cDNA Synthesis kit (Stratagene, La Jolla, Calif., USA) as previously described [19], [20]. Primers TAase (5'-^119^aag/tac/aag/cc(a/c)/ca(a/g)/ga(t/c)/gc^138^-3') and AP (5'-ggccacgcgtcgactagtact-(dt)16-3') were used in the first set of PCR. The product obtained was used as a template in a subsequent PCR with primers TAase and AUAP (5'-ggccacgcgtcgactagtac-3'). The product from the nest PCR reaction was gel purified and inserted into the pGEM-T vector (Promega, Madison, WI, USA) for sequencing analysis.

The full-length cDNA of the *F. proliferatum* transaldolase was obtained by 5' rapid amplification of cDNA end (RACE) reaction. The template cDNA for the reaction was synthesized with reverse transcriptase (RT, Stratagene) and primer GSP-r1 (5'-^529^aagagagaacatgagggtgaggtt^506^-3'). An oligo-(dC) was added to the end of the purified cDNA with terminal deoxynucleotidyl transferase (Promega). Primers GSP-r2 (5'-^336^tcgacctcagttgagacctt^317^-3') and 5R AAP (5'-ggccacgcgtcgactagtacgggiigggiigggiig-3') were then used in the 5'-RACE reaction. The product was purified, subcloned, transformed and subsequently sequenced.

### Preparation of recombinant fungal transaldolases

The *F. proliferatum* transaldolase was expressed with an N-terminal His_6_-tag. Appropriate primers (Fu-TAase-f, 5'-cgggatcc^44^tcttcctctctcgaacagctc^64^-3' and Fu-TAase-r, 5'-aactgcag^1012^ttaggcgagcttctccttgaggatgc^987^-3') were used in the PCR amplification with the full-length cDNA encoding the *F. proliferatum* transaldolase prepared above as template. The PCR products were restricted then ligated into the pQE-80 vector (Qiagen Inc., Valencia, CA, USA) for protein expression in *E. coli* JM109 cells. The recombinant proteins were affinity-purified with Ni-NTA resin columns (Qiagen Inc.) according to the manufacturer’s instructions. The His_6_-tagged recombinant *C. cladosporioides* transaldolase (Cla c 14.0101) used in this study was prepared as described previously [19]. Immuno-reactivity of the recombinant fungal and the recombinant human transaldolases (Novus Biologicals, Littleton, CO, USA; 0.5 µg/strip) against IgE antibodies was analyzed by SDS-PAGE-immunoblot.

### Immunoblot inhibition

For immunoblot inhibition studies, anti-transaldolase IgE-containing serum samples were firstly reacted with purified recombinant *F. proliferatum* transaldolase before incubating with PVDF blots containing *F. proliferatum* extracts, purified rCla c 14.0101, or purified recombinant human transaldolase at 4°C for 16 h. As controls, the blots were incubated with similar serum samples that have been pre-incubated with equivalent amounts of bovine serum albumin (BSA, Pierce, Rockford, IL, USA) or purified recombinant house dust mite allergen Der p 7 [21]. The blots were then washed and incubated with alkaline phosphatase-conjugated monoclonal anti-human IgE antibodies (Pharmingen) and developed with enzyme substrates as described [19], [20]. In addition, two house dust mite-sensitized serum samples were firstly reacted with purified recombinant Der p 7, *F. proliferatum* transaldolase or BSA before incubating with PVDF blots containing Der p 7 at 4°C for 16 h. The blots were then incubated with alkaline phosphatase-conjugated monoclonal anti-human IgE antibodies (Pharmingen) and developed with enzyme substrates as above.

## Results

### Immunoblot reactivity against components of *F. proliferatum*

**Figure 1:**
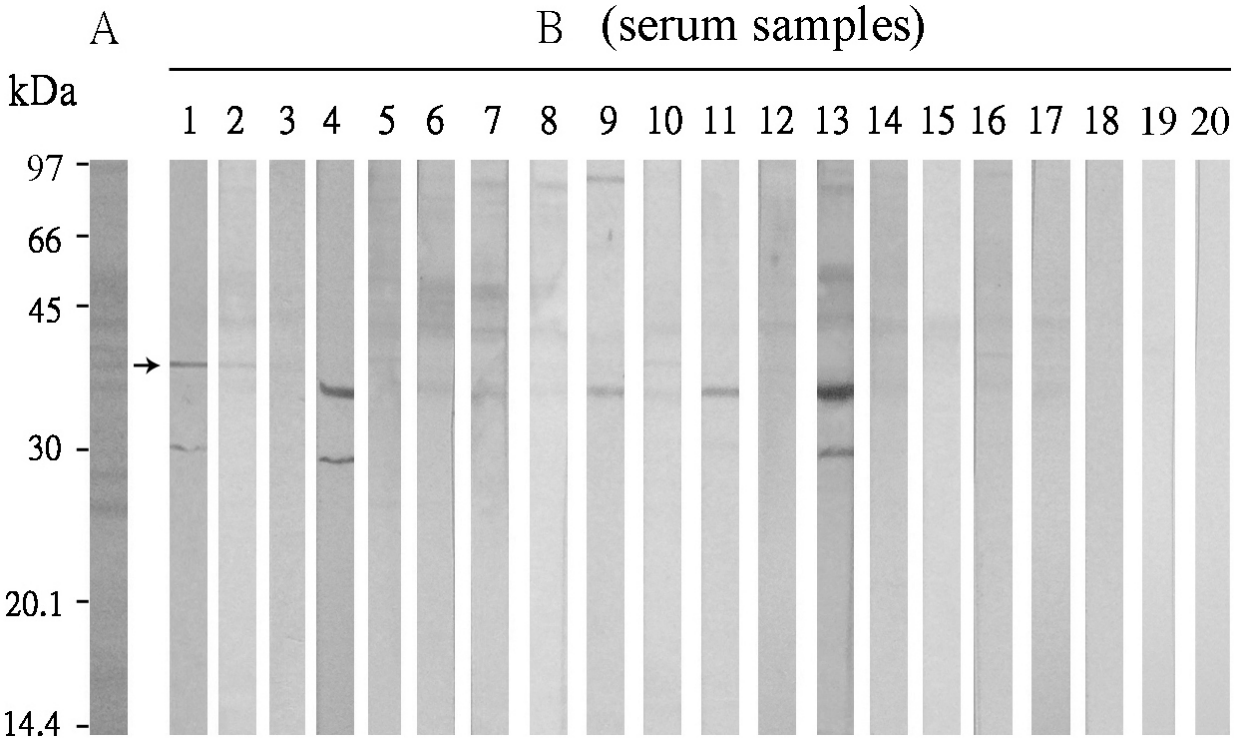
Immunoblot reactivity against components in *F. proliferatum* extracts. (A) Coomassie blue-stained protein profile of *F. proliferatum* extracts. (B) IgE immunoblot profiles obtained with serum samples from respiratory atopic patients (serum nos. 1–17), a non-atopic individual (serum no. 18) and two house dust mite (*D. pteronyssinus*)-sensitized atopic individuals (serum nos. 19, 20). The arrow indicates the position of the 37.5 kDa component of *F. proliferatum.*

*F. proliferatum* crude extracts were separated by SDS-PAGE. The Coomassie blue-stained protein profile of the fungal extracts was shown in panel A of Fig. 1. The separated proteins were blotted onto PVDF membrane then reacted with patient sera. Among the 60 serum samples from respiratory atopic patients examined, 17 (28%) demonstrated IgE-binding against components of *F. proliferatum* (Fig. 1, strip nos. 1–17 of panel B). Human IgE antibodies reacted with at least nine different *F. proliferatum* components ranging in molecular mass from 92 to 30 kDa as shown in Fig. 1 and Table 1. Components of 43 and 36.5 kDa with IgE-binding frequencies of 88% (15/17) and 53% (9/17), respectively, may be considered as major allergens of *F. proliferatum*. In Fig. 1, the 36.5 kDa allergen has a relatively higher intensity of IgE-immunoblot reactivity than others. The 37.5 kDa component (indicated with an arrow, Fig. 1B) with IgE-binding frequency of 47% (8/17) was considered an important allergen of *F. proliferatum*. The 92, 83 and 48 kDa components with IgE-binding frequencies of about 35–41% were considered as significant allergens of *F. proliferatum*. The 40, 32 and 30 kDa components with IgE-binding frequencies of less than 20% were minor allergens of *F. proliferatum*. A serum sample from a non-atopic individual (serum no. 18) and two serum samples from house dust mite (*D. pteronyssinus*)-sensitized atopic individuals (serum nos. 19 and 20) were included as negative controls and shown in Fig. 1, panel B.

**Table 1:** Reactivity of IgE antibodies against components of *F. proliferatum* analyzed by SDS-PAGE-immunoblot.

F. proliferatum		component	Strips of IgE-immunoblot																	
No.	kDa	Frequency of IgE-binding (%)	1	2	3	4	5	6	7	8	9	10	11	12	13	14	15	16	17	18
1	92	41 (7/17)					+*	+	+	+	+				+			+		
2	83	35 (6/17)		+			+	+	+	+					+					
3	48	35 (6/17)		+			+	+	+	+					+					
4	43	88 (15/17)		+	+		+	+	+	+	+	+	+	+	+	+	+	+	+	
5	40	18 (3/17)					+					+						+		
6	37.5	47 (8/17)	+	+	+			+		+	+				+				+	
7	36.5	53 (9/17)				+		+	+	+	+	+	+		+				+	
8	32	12 (2/17)	+										+							
9	30	12 (2/17)				+									+					

*indicates positive IgE-binding as shown in Fig. 1, panel B.

Since a 36.5 kDa IgE-binding component was identified as a transaldolase allergen of *C. cladosporioides* (Cla c 14.0101) [19]. We putatively concluded that the IgE-reacting 37.5 kDa component from *F. proliferatum* was possibly a transaldolase.

### cDNA cloning of the *F. proliferatum* transaldolase

**Figure 2:**
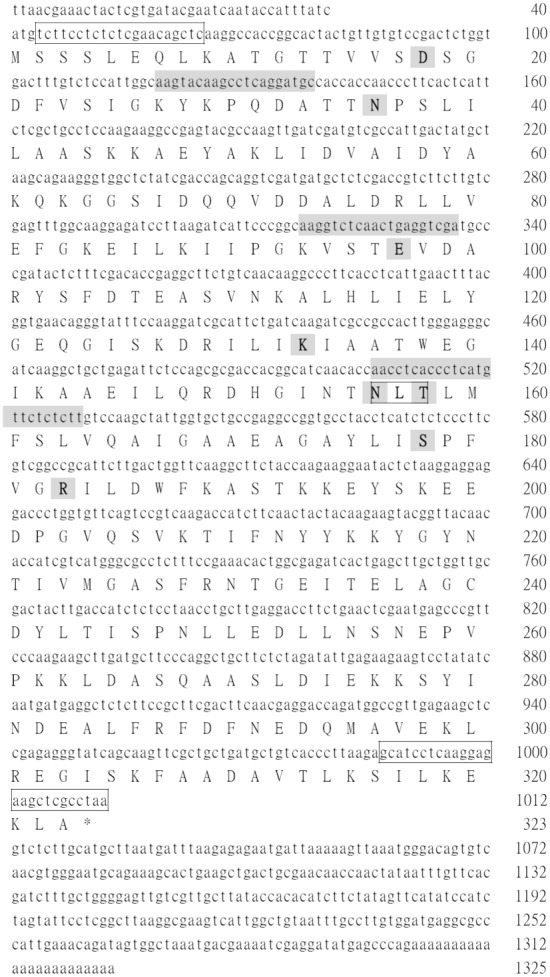
The nucleotide and deduced amino acid sequences of Fus p 4.0101 (GenBank accession no. KF151224). Numbers to the right are the positions of the nucleotides and the deduced residues of the sequences. The stop codon TAA is denoted with an asterisk (*). The potential N-glycosylation site is indicated in bold letters and boxed. Eight conserved amino acid residues that are involved in the catalysis and the substrate binding of transaldolases are shaded and in bold letters. Nucleotide sequences in grey correspond to primer sequences synthesized for PCR experiments in the cDNA cloning of Fus p 4.0101 as described in the Materials and Methods. The sequences corresponding to primers Fu-TAase-f and Fu-TAase-r used in the preparation of rFus p 4.0101 are boxed.

The full-length cDNA encoding the *F. proliferatum* transaldolase was obtained through RT-PCR coupled with the 5'-end RACE reaction. The nucleotide (GenBank accession no. KF151224) and the deduced amino acid sequences of the open reading frame are presented in Fig. 2. A potential polyadenylation signal (AATCGA) for mRNAs of higher eukaryotes was found 15–20 bases upstream from the poly-A tail. The mature *F. proliferatum* transaldolase protein, excluding the initiator methionine [22] has 322 residues and a calculated molecular mass of 35404 daltons, without considering the presence of further post-translational modifications. It has one cysteine (Cys240) and one putative N-glycosylation site (^156^NLT^158^) (Fig. 2). Furthermore, amino acid residues conserved among transaldolases (Asp18, Asn36, Glu97, Lys133, Asn156, Thr158, Ser178 and Arg183) can also be found (Fig. 2). These conserved residues are crucial to enzyme catalysis or substrate binding [23]. This transaldolase allergen has been designated as Fus p 4.0101 by the I.U.I.S. Allergen Nomenclature Sub-committee.

Results from sequence alignment revealed that the *F. proliferatum* transaldolase has 255 (79%), 236 (73%), 247 (77%), 207 (64%) and 195 (61%) amino acids identical to that of *A. fumigatus* (accession no. XM748623), *C. cladoporioides* (accession no. GQ906475), *P. chrysogenum* (accession no. GQ925430), *S. cerevisiae* (accession no. AAB67752), and *Homo sapiens* (accession no. AAF40478) transaldolases, respectively (data not shown). The transaldolase from *F. proliferatum* has only one cysteine residue. However, three cysteine residues are conserved among *A. fumigates, C. cladoporioides* and *P. chrysogenum* transaldolases. In addition, one and two cysteine residues can be found in *S. cerevisiae* and *Homo sapiens* transaldolases, respectively (data not shown). The one potential N-glycosylation site of *F. proliferatum* transaldolase (^156^NLT^158^) is boxed in Fig. 2 and it is conserved among all the transaldolases mentioned above.

### Immunoreactivity of recombinant transaldolase proteins

**Figure 3:**
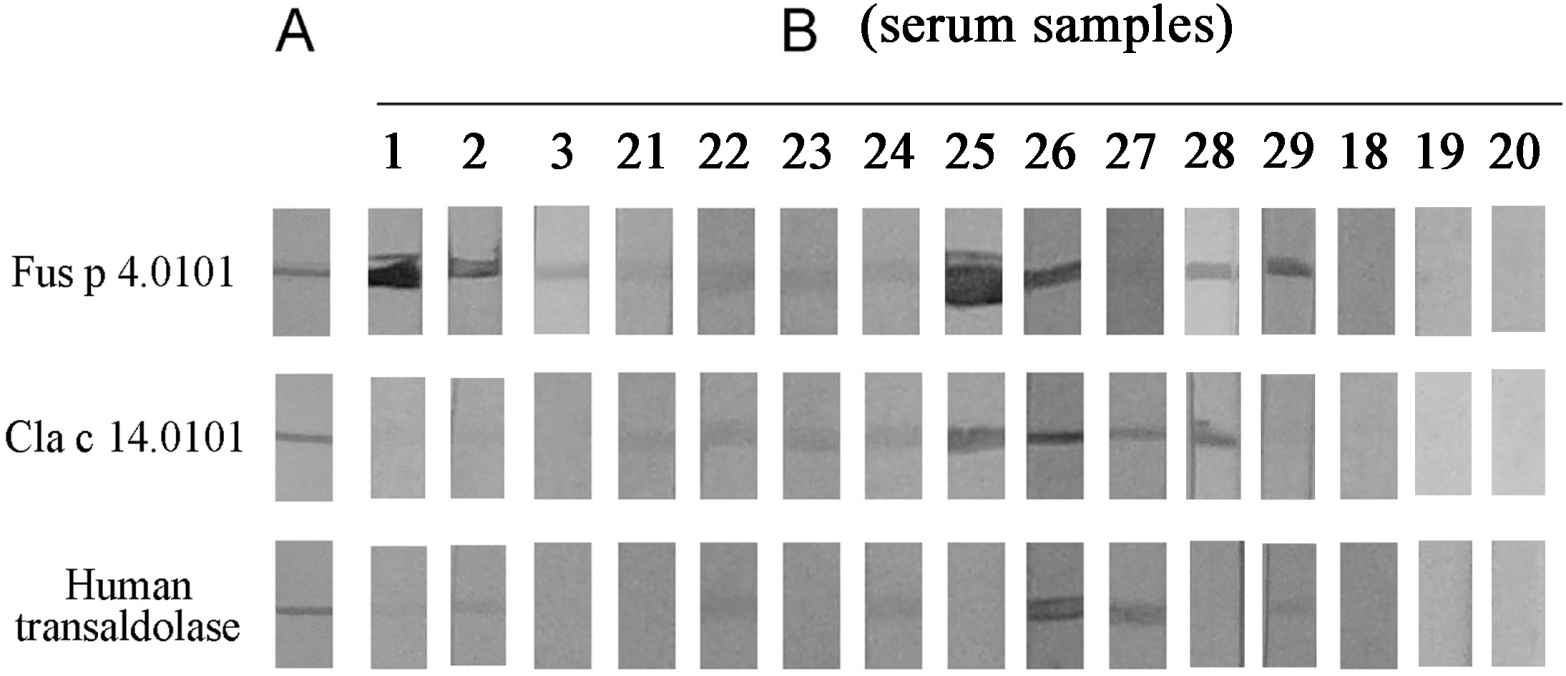
Antigenicity of recombinant *F. proliferatum, C. cladosporioides* and human transaldolases. (A) Coomassie blue-stained protein profile of rFus p 4.0101, rCla c 14.0101 and recombinant human transaldolase on PVDF membranes. (B) IgE immunoblot reactivities of these three recombinant proteins analyzed by using serum samples nos. 1–3 from Fig. 1 (serum nos. 1–3) and nine serum samples from asthmatic patients (serum nos. 21–29) who showed previously IgE-binding reactvities against rCla c 14.0101 or recombinant human transaldolase. Sera from a non-atopic healthy individual (serum no. 18) and two house dust mite-sensitized atopic individuals (serum nos. 19 and 20) were included as controls.

In this study, rFus p 4.0101 was expressed as N-terminal His_6_-tag proteins in *E.coli* and purified (Fig. 3, panel A). It has an apparent molecular mass of about 38 kDa upon SDS-PAGE analysis (data not shown). The Coomassie blue-stained protein profiles of rFus p 4.0101, rCla c 14.0101 and a recombinant human transaldolase obtained commercially are shown in Fig. 3, panel A. Serum sample nos. 1–3 from Fig. 1, panel B showed positive IgE-binding against rFus p 4.0101. These three serum samples have negative IgE-binding to rCla c 14.0101 and serum no. 2 showed positive IgE-binding against human transaldolase (Fig. 3, panel B). In addition, nine serum samples from asthmatic patients with IgE-binding against rCla c 14.0101 or recombinant human transaldolase [19] were included in this study. The IgE immunoblot reactivities of these sera (serum nos. 21–29) against these three different recombinant transaldolases are shown in Fig. 3, panel B. Among these nine serum samples, eight (serum nos. 21–26, 28 and 29) showed positive IgE-binding against rFus p 4.0101. Eight (serum nos. 21–28) of these nine serum samples showed IgE-binding against rCla c 14.0101. Furthermore, serum nos. 22, 24, 26, 27 and 29 have also IgE reactivity against recombinant human transaldolase. Serum from a non-atopic individual (serum no. 18) and from two house dust mite-sensitized atopic individuals (serum nos. 19 and 20) were used as controls and showed negative IgE immunoblot reactivity against all three recombinant transaldolases (Fig. 3, panel B).

### Immunoblot inhibition

**Figure 4:**
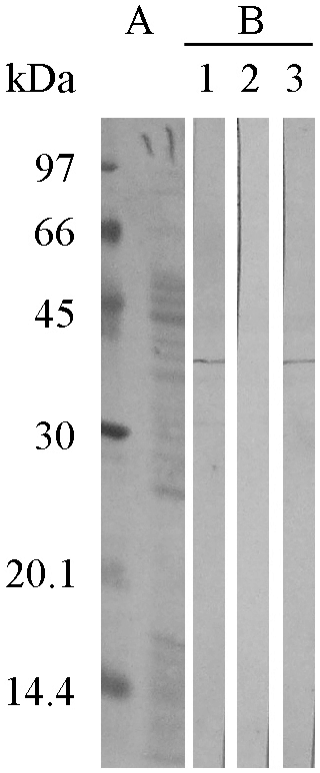
Immunoblot inhibition of IgE-binding against nFus p 4.0101 in crude *F. proliferatum* extracts with purified rFus p 4.0101 and BSA as inhibitors. (A) Coomassie blue-stained protein profile of *F. proliferatum* extracts and protein molecular weight markers. (B) IgE binding against the 37.5 kDa component using serum no. 1 from figure 1B (lane 1); this binding activity was inhibited with 10 µg of rFus p 4.0101 (lane 2) but not BSA (lane 3).

The relationship between rFus p 4.0101 and the IgE-binding 37.5 kDa component of *F. proliferatum* was further delineated with immunoblot inhibition. Fig. 4 showed that serum sample no. 1 from panel B of Fig. 1 has IgE immunoblot reactivity against the 37.5 kDa component of *F. proliferatum* (Fig. 4, strip 1 of panel B). This immunoblot reactivity was inhibited when the same serum sample was pre-absorbed with 10 µg of rFus p 4.0101 (Fig. 4, strip 2 of panel B). Pre-absorption of the serum sample with 10 µg of BSA did not inhibit its IgE binding against the 37.5 kDa component of *F. proliferatum* (Fig. 4, strip 3 of panel B). Results obtained correlate the 37.5 kDa component with the *F. proliferatum* transaldolase and much of the IgE determinants on the native transaldolase were conserved in the recombinant Fus p 4.0101.

### Immunoglobulin E cross-reactivity

**Figure 5:**
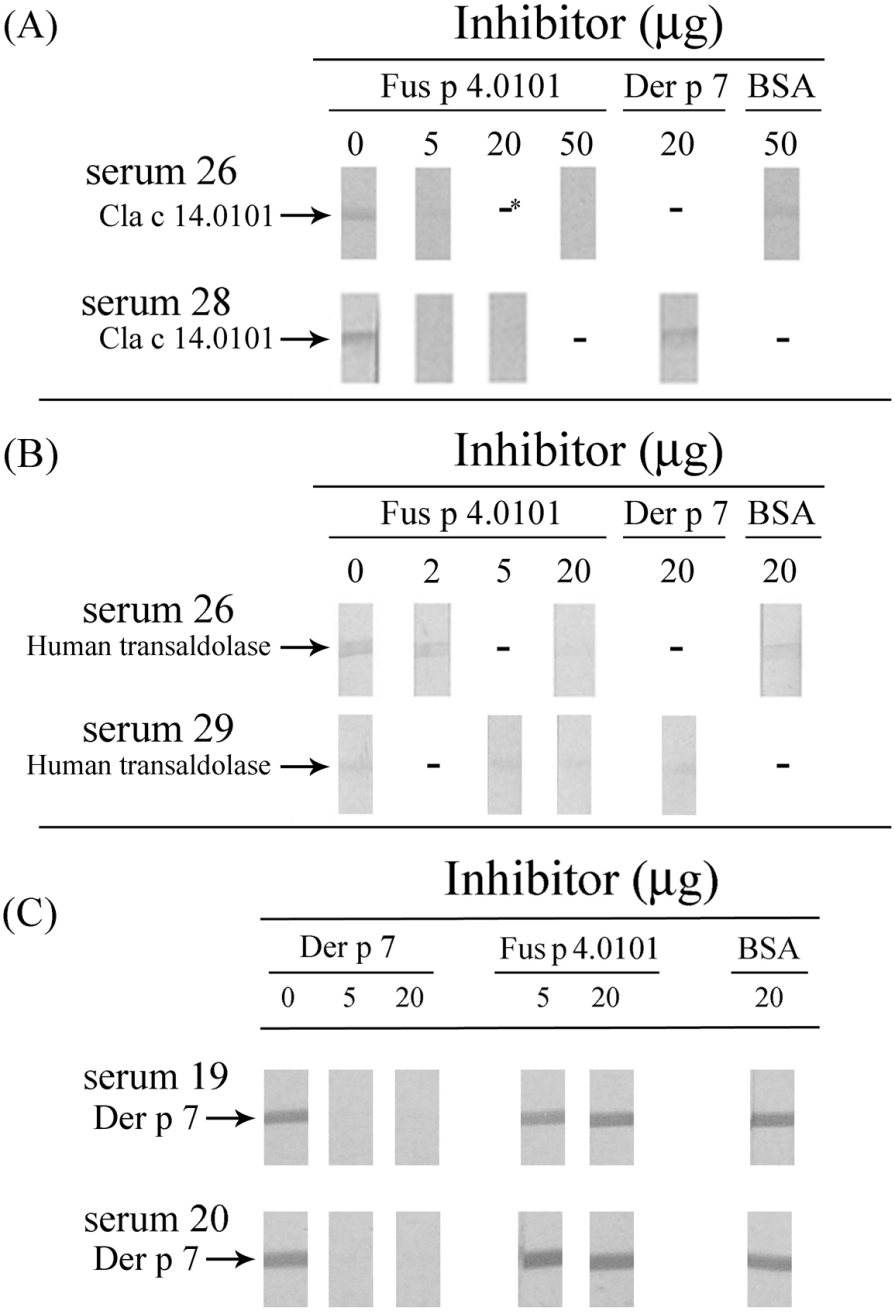
Inhibition of IgE-immunoblot reactivity against (A) rCla c 14.0101, (B) recombinant human transaldolase and (C) Der p 7. IgE-immunoblot experiments were carried out with serum nos. 19, 20, 26, 28 and 29 from Fig. 3. Serum was pre-absorbed with the amount of rFus p 4.0101, Der p 7 or BSA as indicated. * indicates not done.

The panel A of Fig. 5 showed that serum nos. 26 and 28 from Fig. 3, panel B has IgE-binding activity against rCla c 14.0101. rFus p 4.0101 can inhibit, dose dependently, this reactivity (Fig. 5, panel A). Pre-absorption of serum sample no. 26 with 50 µg of BSA or pre-absorption of serum sample no. 28 with 20 µg of rDer p 7 did not inhibit its IgE binding against rCla c 14.0101 (Fig. 5, panel A). The results suggest IgE cross-reactivity between transaldolases from *Fusarium* and *Cladosporium* fungi.

Serum sample no. 26 has also IgE-binding activity against recombinant human transaldolase (Fig. 3, panel B and Fig. 5, panel B). This activity can be inhibited dose dependently by pre-absorbing the serum with 2 or 20 µg of rFus p 4.0101. Inhibition of IgE binding was not detected when the same serum was pre-absorbed with 20 µg of BSA (Fig. 5, panel B). In addition, the IgE-binding activity against recombinant human transaldolase of serum no. 29 can also be inhibited dose dependently by pre-absorbing the serum with 5 or 20 µg of rFus p 4.0101 (Fig. 5, panel B). Inhibition of IgE binding was not detected when the same serum was pre-absorbed with 20 µg of Der p 7 (Fig. 5, panel B).

Results in Fig. 5, panel C demonstrated that IgE-binding against recombinant Der p 7 in two house dust mite-sensitized atopic serum samples (serum nos. 19 and 20 in Fig. 1 and Fig. 3) can be inhibited by pre-absorbing both serum samples with 5 or 20 µg of rDer p 7. But similar amounts of Fus p 4.0101 or BSA cannot inhibit this binding activity.

## Discussion

Fungi are prominent sources of allergens. However, they are still largely neglected in clinical practice and basic research [24]. Allergens of the frequent mold genera including *Cladosporium*, *Alternaria*, *Aspergillus* and *Penicillium* have been characterized and reported [2], [3], [18]–[20], [24]. *Fusarium* is among airborne fungi that contribute to human respiratory atopic disorders worldwide. It is important to well characterize IgE-recognizing *Fusarium* components. In this study, among 60 respiratory atopic sera tested, 17 (28%) demonstrated IgE-binding against nine different components from *F. proliferatum*. The nature of the 43 and 36.5 kDa major *F. proliferatum* allergens is currently under investigation and will be published separately (Shen et al., manuscript in preparation). The 37.5 kDa component of *F. proliferatum* has an IgE-binding frequency of 47% (8/17). IgE-binding against this 37.5 kDa component can be inhibited by a recombinant *F. proliferatum* transaldolase (Fig. 4). Our results indicate that this important 37.5 kDa IgE-binding component is a *F. proliferatum* transaldolase.

Allergens of the *Fusarium* species have been reported by three different groups. Verma et al. [16] reported from India that the culture filtrates from *F. solani* contained 18 allergenic proteins as determined by immunoblotting. A 65-kDa protein component reacted with IgE antibodies in all 15 patient sera tested and was considered as a major allergen [16]. The 45 and 14 kDa components reacted against IgE in 12 patients’ sera. The 41, 38, 35 and 30 kDa *F. solani* components reacted with IgE antibodies in 9 of the 15 patients’ sera tested [16]. In studies of allergens of *F. equiseti*, a 65 kDa protein was also found as a major allergen of this common *Fusarium* species [25]. The nature of the 65 kDa major allergen needs further study. The 45 kDa *F. solani* allergen has an N-terminal sequence of Lys-Gly-Arg-Thr-Glu-Phe-Ala, which does not show homology to any known fungal proteins [26]. Through cDNA cloning, Hoff et al. in Europe identified three *F. culmorum* allergens (Fus c 1, Fus c 2 and Fus c 3) [13]. The 11 kDa Fus c 1 (60S acidic ribosomal protein P2), 13 kDa Fus c 2 (thioredoxin-like protein) and 49 kDa Fus c 3 (not related to known proteins) have IgE-binding frequencies of 35, 50, and 15%, respectively, with sera from 26 individuals sensitized to *F. culmorum* [13]. Recently, Khosravi et al. in Iran showed with immunoblotting that *F. solani* has six major allergens with molecular masses of 24, 58.5, 64.5, 69, 72 and 97 kDa [27]. In this study, we did not detect a major IgE-binding *F. proliferatum* protein with molecular mass of about 65 kDa. Whether our 37.5 and 43 kDa IgE-reacting *F. proliferatum* components correspond to the 38 and 45 kDa *F. solani* allergens reported need further clarification. Furthermore, we did not detect low molecular mass IgE-binding *F. proliferatum* proteins correlate to the 11 and 13 kDa *F. culmorum* allergens. Whether our 48 kDa IgE-reacting *F. proliferatum* protein resembles the 49 kDa *F. culmorum* allergen also needs further elucidation. The utilization of different fungal strains, the variations in the culturing conditions, the variance of the methods used in preparing the fungal extracts, and the divergences in exposure and genetic background of individuals examined all contribute to discrepancies in results obtained from various studies and research groups [4]. Thus, studies of fungal allergens at molecular level to provide a basis for standardized fungal extracts is of major importance in clinical allergy.

Fungal-atopic subjects often demonstrated a parallel and independent multiple sensitization to many different fungal species [13], [17]. Patients may be sensitized by allergens from individual fungal species. Their multiple sensitizations could be caused by IgE cross-reactivity of a particular protein common among various fungal species. We have observed IgE cross-reactivity among the vacuolar serine protease major pan-fungal allergens [18], [20]. Our results here indicate that the 37.5 kDa transaldolase is an important allergen of *F. proliferatum*. Transaldolases have been identified as important allergens of prevalent airborne *Cladosporium* (Cla c 14.0101) and *Penicillium* (Pen c 35) species [19]. IgE cross-reactivity between Cla c 14.0101 and Pen c 35 has been demonstrated [19]. Transaldolase allergens from *Cladosporium*, *Penicillium* and *Fusarium* species share 67% (217/323 residues) amino acid sequence identity. Our results in Fig. 3 showed that seven sera have IgE reactivity against both Fus p 4.0101 and Cla c 14.0101 and suggested IgE cross-reactivity exists between the *Fusarium* and *Cladosporium* transaldolase allergens. Results from IgE-immunoblot inhibition (Fig. 5, panel A) with serum nos. 26 and 28 in Fig. 3 confirmed the presence of IgE cross-reactivity between Fus p 4.0101 and Cla c 14.0101. Combining with previous results [19], it is suggestive that IgE cross-reactivity prevails among the transaldolase allergens from *Fusarium*, *Penicillium* and *Cladosporium* species. Similarly, Verma et al. detected allergenic cross-reactivity among the 14 kDa protein component of three different *Fusarium* species [28]. In addition, the 45 kDa *F. solani* major allergen has allergenic cross-reactivity with fungal extracts prepared from *Alternaria, Cladosporium, Curvularia* and *Epicocum* species [26]. Furthermore, Hoff et al. also demonstrated IgE cross-reactivity between *F. culmorum* and *A. alternata* allergens [13]. All these results provide important information in clinical fungal allergy.

Transaldolase from *F. proliferatum, C. cladosporioides* and *homo sapiens* share 54% (173/322 residues) amino acid sequence identity. Interestingly, five of the eleven rFus p 4.0101 IgE-positive sera (serum nos. 2, 22, 24, 26 and 29) and one of the rCla c 14.0101 IgE-positive serum (serum no. 27) showed IgE-binding to recombinant human transaldolase (Fig. 3, panel B). Similarly, among another eight fungal (Cla c 14.0101) transaldolase-positive sera tested previously, three of them showed IgE-binding against the recombinant human transaldolase [19]. In this study, IgE cross-reactivity between *Fusarium* and human transaldolase was further demonstrated (Fig. 5, panel B). In literature, evolutionarily conserved IgE-reactive human antigens corresponding to fungal and pollen allergens such as profilin, ribosomal P2 protein, and manganese superoxide dismutase proteins have been reported [29]–[31]. Recognition of these human counterparts by IgE antibodies might contribute to the stimulation of type I hypersensitive reactions in the absence of exogenous allergen exposure [30]. It might also play a role in certain chronic and severe allergic disorders [29], [31]. Results obtained from this study provide further evidence in IgE cross-reactivity between an environmental fungal allergen and its human analogue which might contribute to disease manifestations.

In conclusion, we identified an important novel transaldolase allergen of *F. proliferatum*. In addition, IgE cross-reactivities between *Fusarium* and *Cladosporium* transaldolase allergens as well as between *Fusarium* and human transaldolases were demonstrated. Our results provide important information in clinical fungal allergy.
